# ASSOCIATION OF MASSETER MUSCLE VOLUME WITH RISK OF OSTEOPOROSIS IN OLDER ADULTS: THE BUNKYO HEALTH STUDY

**DOI:** 10.1093/geroni/igad104.3194

**Published:** 2023-12-21

**Authors:** Abulaiti Abudurezake, Saori Kakehi, Shota Sakamoto, Minami Morikawa, Hideyoshi Kaga, Yoshifumi Tamura, Hiroki Tabata, Ryuzo Kawamori

**Affiliations:** Juntendo University Graduate School of Medicine, Bunkyo-Ku, Tokyo, Japan; Juntendo University Graduate School of Medicine, Bunkyo-Ku, Tokyo, Japan; Juntendo University Graduate School of Medicine, Bunkyo-Ku, Tokyo, Japan; Juntendo University Graduate School of Medicine, Bunkyo-Ku, Tokyo, Japan; Juntendo University Graduate School of Medicine, Bunkyo-Ku, Tokyo, Japan; Juntendo University Graduate School of Medicine, Bunkyo-Ku, Tokyo, Japan; Juntendo University Graduate School of Medicine, Bunkyo-Ku, Tokyo, Japan; Juntendo University Graduate School of Medicine, Bunkyo-Ku, Tokyo, Japan

## Abstract

Abstract The relationship between bone mineral density (BMD) and oral function has been previously studied. However, the relationship between the masseter muscle, which plays an important role in oral function, and BMD and osteoporosis in older adults has not been investigated. This study investigated the association between masseter muscle volume (MMV) and old age in relation to osteoporosis prevalence and hip joint BMD. Body composition and BMD of 1534 people aged 65–84 years living in Bunkyo-ku, Tokyo, were measured using dual-energy X-ray absorptiometry. MMV was measured using a three-dimensional medical image analysis system. Participants were divided into five groups based on MMV by 20% for each gender (Quintile 1–5: Q1–5, Q1< Q5). Logistic regression models were employed to estimate multivariable-adjusted odds ratios (ORs) for osteoporosis determined by T-score (<−2.5 standard deviation) using the Q5 reference. Subsequently, analysis of covariance was used to investigate the relationship between MMV and BMD. Adjustment for age, body mass index, smoking history, alcohol intake, calcium intake, 25(OH)D, diabetes, and osteoporosis drugs or estrogen. In men, Q1 had higher hip osteoporosis prevalence than Q5 (OR 11.65; 95% confidence interval, 1.48–1.62, p=0.020). Further, hip BMD was significantly higher in Q5 than in Q1 (p=0.001). In women, MMV and old age was not associated with osteoporosis prevalence. However, hip BMD was significantly higher in Q3,4 than in Q1 (p=0.003). Older adults with lower MMV had lower BMD in the hip joint. Additionally, men had higher odds of osteoporosis of the hip joint.

